# Sequential Varicella and Herpes Zoster in an Immunocompetent Adult

**DOI:** 10.1002/ccr3.72174

**Published:** 2026-02-24

**Authors:** Bibek Sharma, Supriya Paudel

**Affiliations:** ^1^ Department of Dermatology Madan Bhandari Academy of Health Sciences Hetauda Nepal; ^2^ Department of Dermatology Madan Bhandari Academy of Health Sciences Hetauda Nepal

**Keywords:** herpes zoster, sequential, varicella, varicella‐zoster virus

## Abstract

Varicella and herpes zoster, caused by primary infection and reactivation of varicella‐zoster virus (VZV) respectively, can present with a wide spectrum of cutaneous and systemic complications. This case highlights an uncommon sequential presentation of varicella and zoster in an immunocompetent adult in noncontiguous dermatomes and emphasizes the importance of clinicopathological correlation in atypical presentations.

## 1 Introduction

Varicella and herpes zoster are distinct clinical entities caused by varicella‐zoster virus (VZV). Primary infection manifests as varicella, while herpes zoster occurs due to viral reactivation from sensory ganglia, usually decades later. The risk of herpes zoster increases with age and immunosuppression [1]. Sequential or near‐simultaneous occurrence of varicella and herpes zoster in immunocompetent adults is exceptionally rare, with only a few cases reported in the literature [2, 3]. Here, we present such a case in a 45‐year‐old male, with clinicopathological confirmation and unusual cervical dermatomal involvement.

## 2 Case History

**FIGURE 1:**
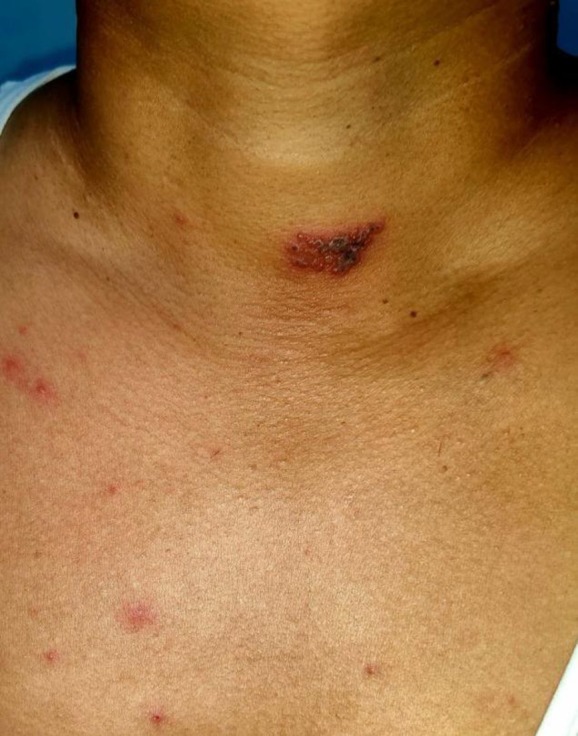
Grouped vesicles on left C4 dermatome.

**FIGURE 2:**
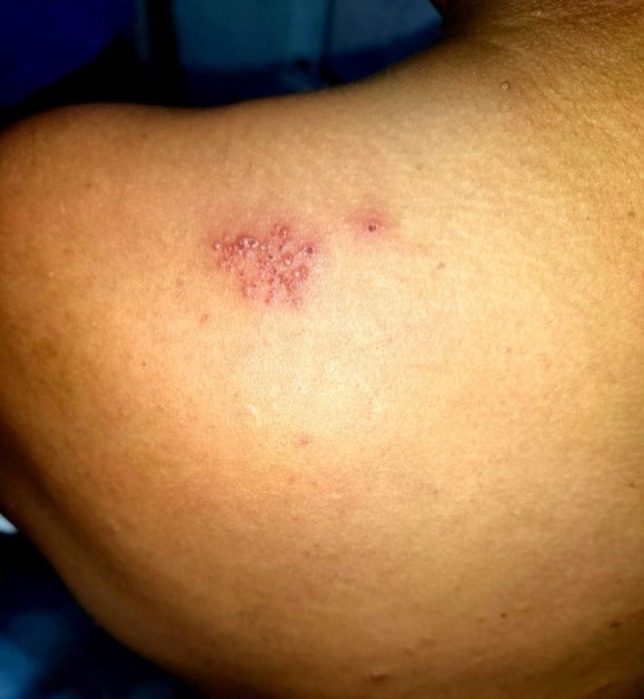
Grouped vesicles on left C7 dermatome.

**FIGURE 3:**
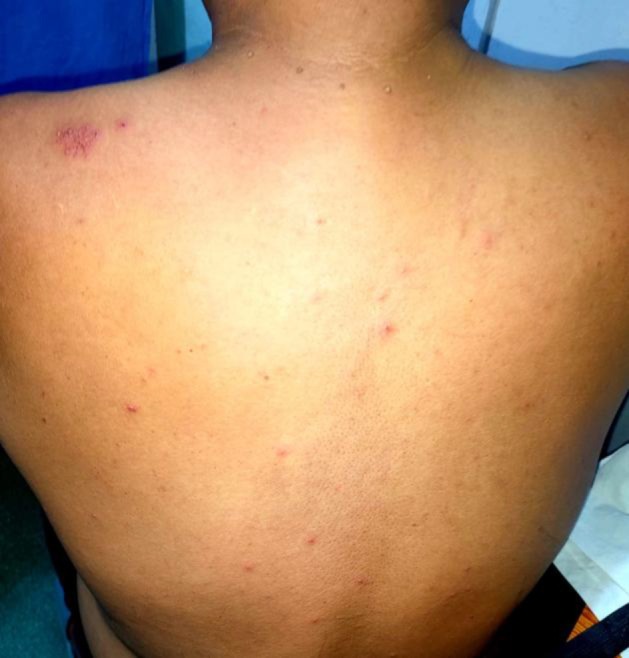
Varicella lesions on the trunk.

A 45‐year‐old previously healthy male with no known comorbidities presented with acute febrile illness followed by cutaneous eruptions. The patient initially developed fever and myalgia, followed 2 days later by generalized pleomorphic vesicles consistent with varicella. Within three subsequent days, he developed severe shoulder pain on the left side. On examination, the trunk and extremities showed pleomorphic papules, vesicles, and crusted lesions (Figure 1) In addition, grouped vesicles on an erythematous base were distributed along the left C4 and C7 dermatomes, suggestive of herpes zoster (Figures 2 and 3).

## 3 Methods

**FIGURE 4:**
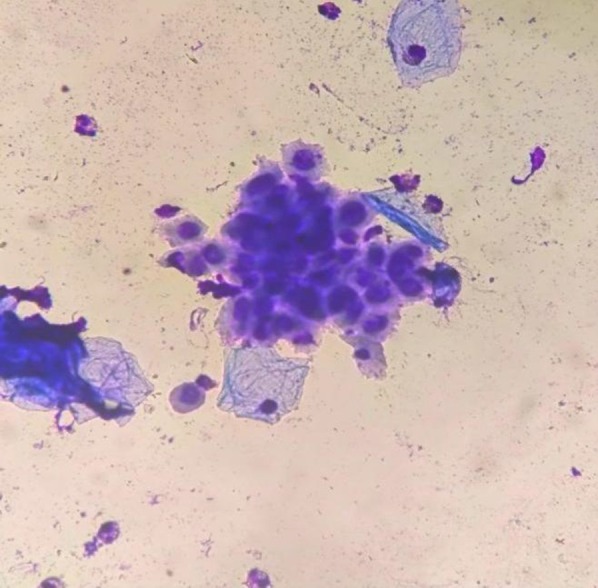
Giemsa stain showing multinucleated giant cells with ballooning degeneration (×400).

Laboratory investigations, including complete blood count, liver and renal function, fasting glucose, and HIV serology, were within normal limits. Giemsa stain of vesicular fluid revealed multinucleated giant cells with ballooning degeneration, consistent with herpesvirus cytopathic changes [4] (Figure 4).

He was treated with oral acyclovir 800 mg five times daily for 7 days; the lesions improved significantly, with complete resolution and no postherpetic neuralgia on follow‐up [5].

## 4 Conclusion and Results

The sequential development of varicella and herpes zoster in an immunocompetent adult is extremely uncommon as most zoster cases occur years to decades after primary infection [1].

## 5 Discussion

Several nonmutually exclusive mechanisms may explain this phenomenon. First, transient immune dysregulation induced by acute primary VZV infection or by other concurrent stress could permit a window for premature viral reactivation from sensory ganglia [3, 6]. Second, simultaneous exposure to distinct VZV strains or infection with multiple viral genotypes has been proposed in older case reports, and recent molecular studies confirmed different or vaccine‐strain sequences underlying atypical presentations. Where available, PCR and genotype analysis can help distinguish reactivation from coinfection [2, 6]. Third, true concurrent reactivation with other herpesviruses (e.g., HSV and VZV) has been described, highlighting the broader phenomenon of herpesvirus coreactivation in certain clinical settings [7].

Our patient demonstrated involvement of noncontiguous cervical dermatomes (C4 and C7). While thoracic dermatomes are most frequently affected in herpes zoster, cervical involvement, particularly multidermatomal, has been described and is often associated with severe neuropathic pain and risk of postherpetic neuralgia [8]. Early recognition is crucial to avoid misdiagnosis, particularly when zoster lesions appear atypically during or shortly after varicella. Previous reports suggest that early initiation of antiviral therapy, such as acyclovir, significantly reduces morbidity and prevents complications, even in immunocompetent adults. He received a 7‐day standard acyclovir course, consistent with international recommendations, leading to resolution without complications [5]. The addition of Giemsa cytology, demonstrating multinucleated giant cells, provided histopathological evidence, further strengthening the diagnosis.

Our case contributes to the limited literature on sequential varicella and herpes zoster in an immunocompetent adult with cervical noncontiguous dermatomal involvement, highlighting the possible molecular mechanism and importance of clinicopathological correlation, especially when faced with atypical presentations.

## Author Contributions

**Bibek Sharma:** conceptualization, formal analysis, resources, software, validation, visualization, writing – original draft, writing – review and editing. **Supriya Paudel:** conceptualization, formal analysis, resources, supervision, validation, visualization, writing – original draft.

## Funding

The authors have nothing to report.

## Consent

A written informed consent was obtained from the patient to publish this report in accordance with the journal's patient consent policy.

## Conflicts of Interest

The authors declare no conflicts of interest.

## Data Availability

The data that support the findings of this study are openly available in [Figshare] at http://doi.org/[10.1002/ccr3.72174], reference number [72174].
